# Supplementary material for: Erastin Inhibits Septic Shock and Inflammatory Gene Expression via Suppression of the NF-κB Pathway
Source: J Clin Med. 2019 Dec 14;8(12):2210. doi: 10.3390/jcm8122210 (PMC6947339; doi:10.3390/jcm8122210)
Supplement: Supplementary file 1 [file jcm-08-02210-s001.pdf]

Journal of
Clinical Medicine

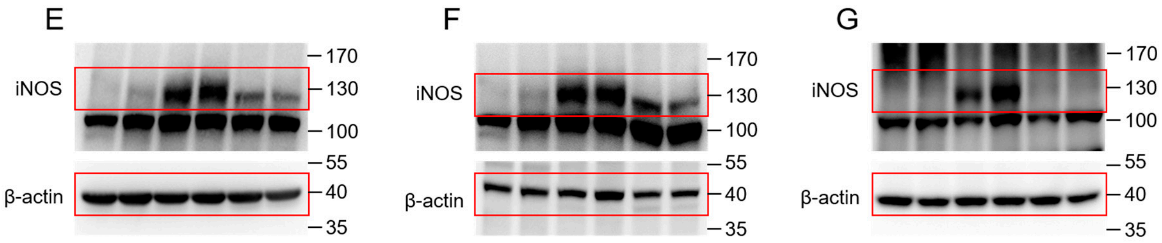

Figure S1. Uncropped images of blots presented in the main Figures 2.

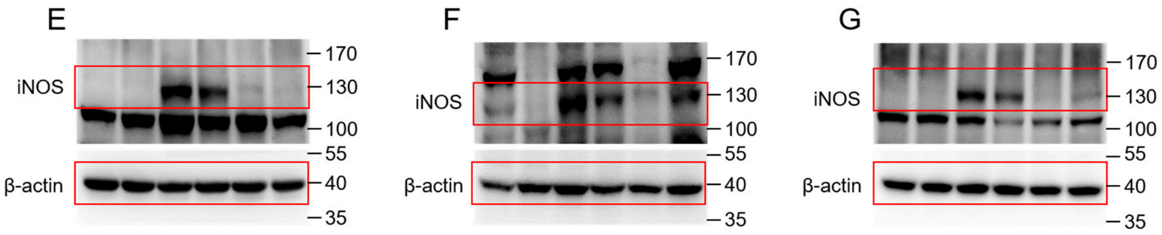

Figure S2. Uncropped images of blots presented in the main Figures 3.

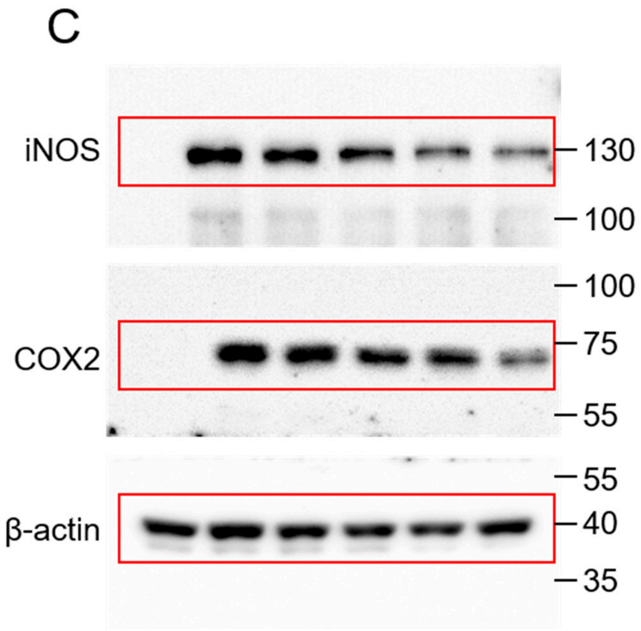

Figure S3. Uncropped images of blots presented in the main Figures 4.

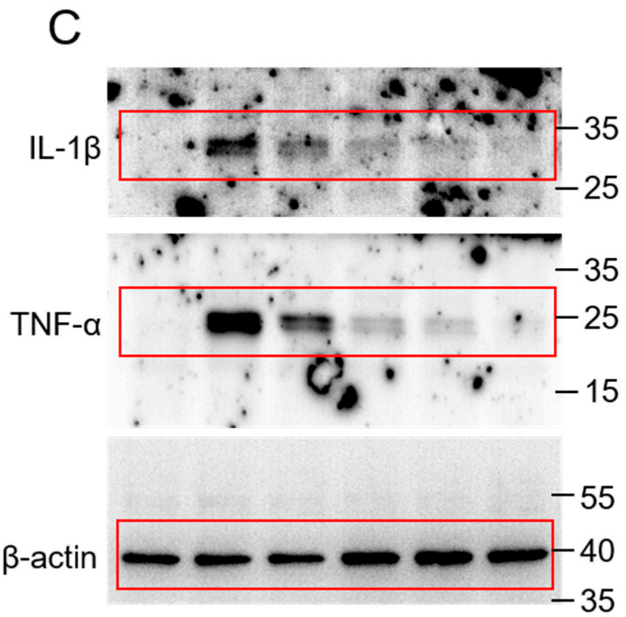

Figure S4. Uncropped images of blots presented in the main Figures 5.

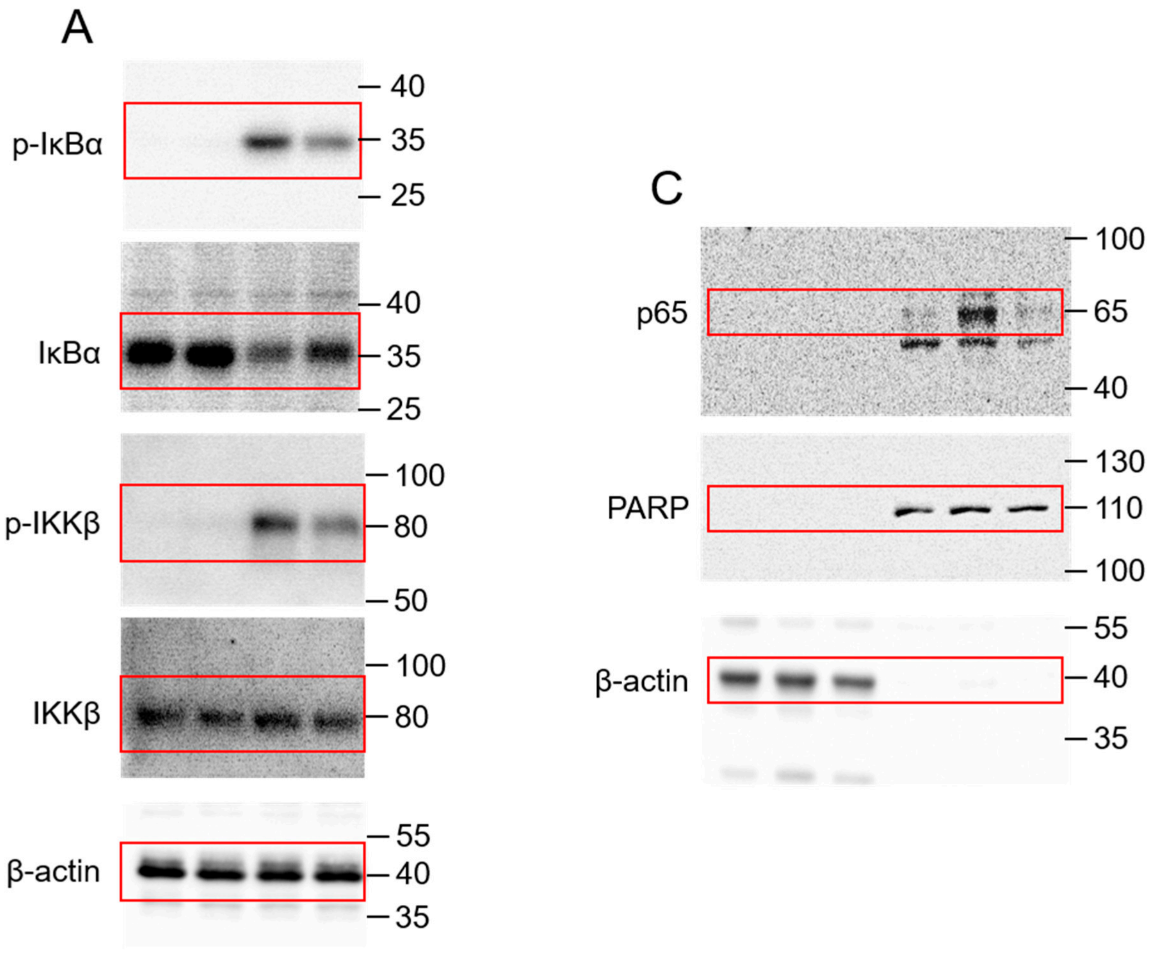

Figure S5. Uncropped images of blots presented in the main Figures 6.

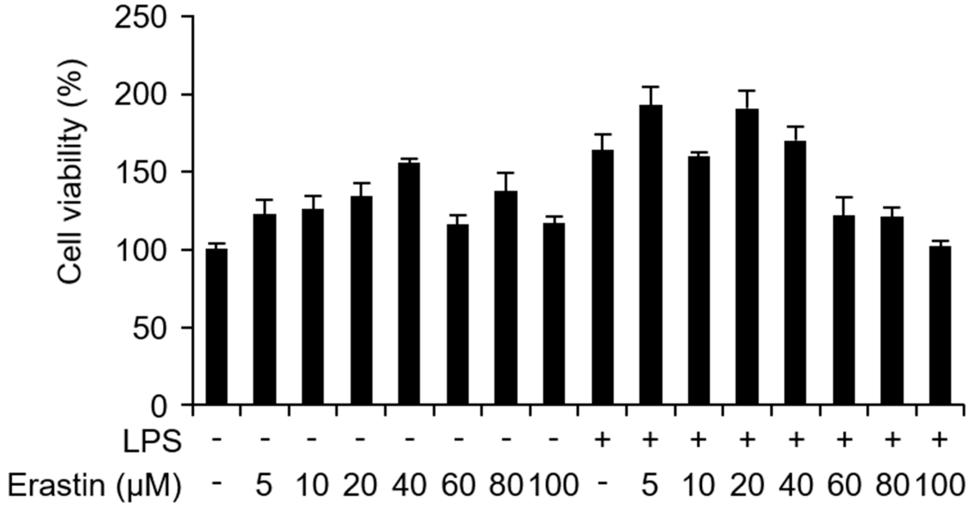

Figure S6. Measuring of erastin-induce cytotoxicity in bone marrow-derived macrophages (BMDMs). Cell viability assay was conducted to determine the dose-dependent effects of erastin in bone marrow-derived macrophages (BMDMs). BMDMs were cultured for 24h with indicated does of erastin with or without 1 μg/ml of LPS stimulation.

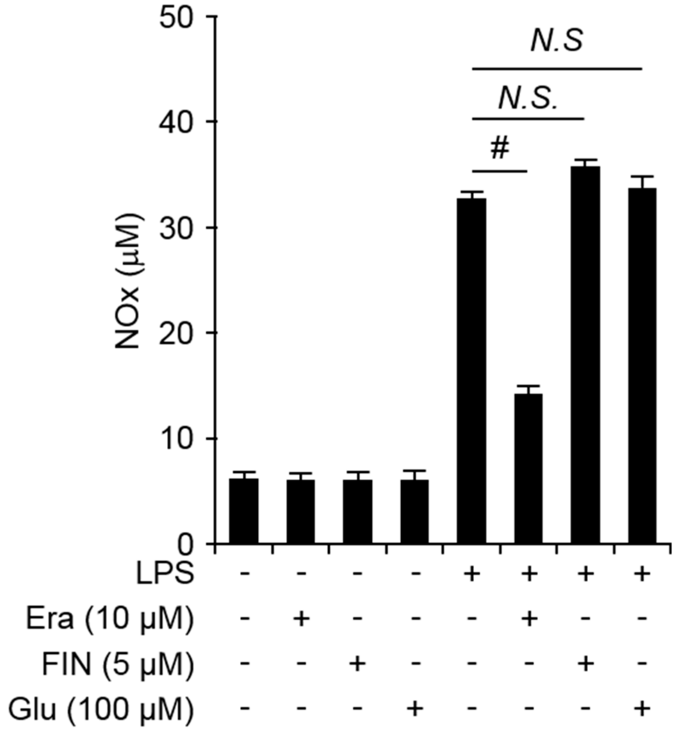

Figure S7. FIN65 and glutamic acid, do not exert anti-inflammatory effects. Nitric oxide (NO) metabolite secretion was measured after incubation with 1 μg/ml of LPS for 24 h with or without various ferroptosis inducers such as erastin (Era), FIN56 (FIN), and glutamic acid (Glu) in bone marrow-derived macrophages (BMDMs) at the indicated dose. Statistical analyses were performed by the paired two-tailed Student's *t*-test. $^{*}p < 0.05$, $^{\#}p < 0.01$.

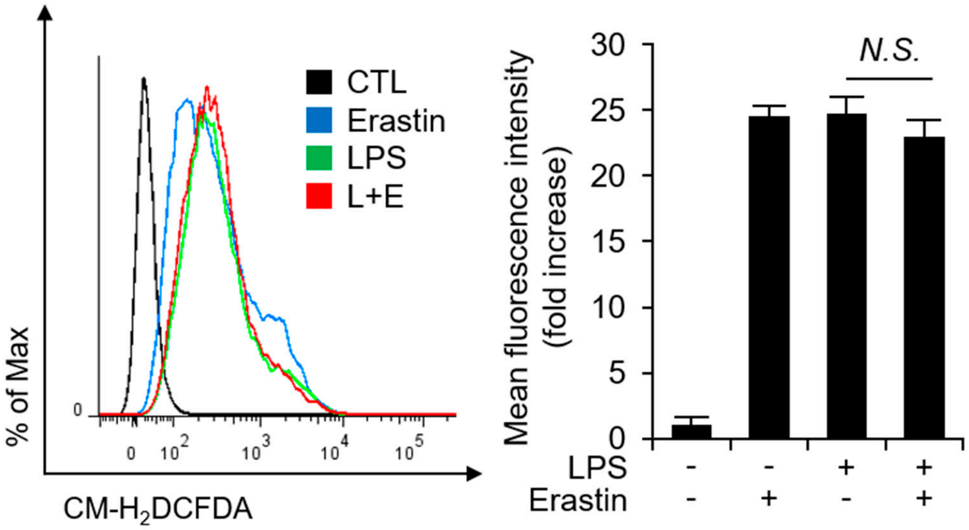

Figure S8. LPS and erastin promotes the production of intracellular reactive oxygen species (ROS). BMDMs were treated with 1 ug/ml of LPS for 2h in the presence or absence of DMSO or 20uM erastin. Cells were stained with CM-H2DCFDA to detect the production of ROS. Graph represents mean fluorescence intensity of three independent experiments.

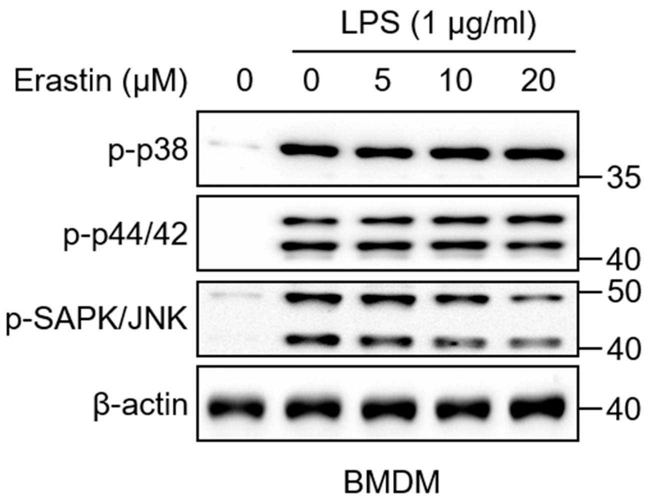

Figure S9. Erastin does not affect LPS-induced MAPK activation in BMDMs. Western blot analysis of p-p38, p-p44/42, and p-SAPK/JNK after pre-incubation with or without erastin at the indicated dose for 2 h and following stimulation with 1 μg/ml of LPS for 1 h using bone marrow-derived macrophages (BMDMs); β-actin was used as a loading control.

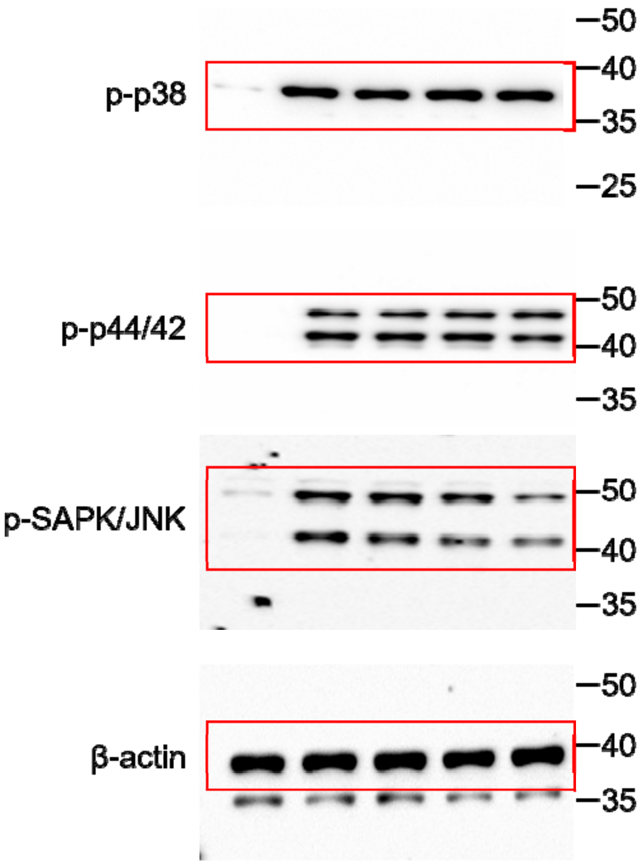

Figure S10. Uncropped images of blots presented in the main Supplementary Figures 3.
